# Active matter in space

**DOI:** 10.1038/s41526-022-00230-7

**Published:** 2022-11-24

**Authors:** Giorgio Volpe, Clemens Bechinger, Frank Cichos, Ramin Golestanian, Hartmut Löwen, Matthias Sperl, Giovanni Volpe

**Affiliations:** 1 Department of Chemistry, University College London, 20 Gordon Street, WC1H 0AJ London, United Kingdom; 2 Physics Department, University of Konstanz, 78457 Konstanz, Germany; 3 Peter Debye Institute for Soft Matter Physics, Faculty of Physics and Earth Sciences, Leipzig University, 04103 Leipzig, Germany; 4 Max Planck Institute for Dynamics and Self-Organization (MPI-DS), 37077 Göttingen, Germany; 5 Rudolf Peierls Centre for Theoretical Physics, University of Oxford, Oxford, OX1 3PU United Kingdom; 6 Institut für Theoretische Physik II: Weiche Materie, Heinrich-Heine-Universität Düsseldorf, Universitätsstrasse 1, 40225 Düsseldorf, Germany; 7 Institut für Materialphysik im Weltraum, Deutsches Zentrum für Luft- und Raumfahrt (DLR), 51170 Köln, Germany; 8 Physics Department, University of Gothenburg, 41296 Gothenburg, Sweden

**Keywords:** Structure of solids and liquids, Soft materials, Biophysics, Structure of solids and liquids, Colloids

## Abstract

In the last 20 years, active matter has been a highly dynamic field of research, bridging fundamental aspects of non-equilibrium thermodynamics with applications to biology, robotics, and nano-medicine. Active matter systems are composed of units that can harvest and harness energy and information from their environment to generate complex collective behaviours and forms of self-organisation. On Earth, gravity-driven phenomena (such as sedimentation and convection) often dominate or conceal the emergence of these dynamics, especially for soft active matter systems where typical interactions are of the order of the thermal energy. In this review, we explore the ongoing and future efforts to study active matter in space, where low-gravity and microgravity conditions can lift some of these limitations. We envision that these studies will help unify our understanding of active matter systems and, more generally, of far-from-equilibrium physics both on Earth and in space. Furthermore, they will also provide guidance on how to use, process and manufacture active materials for space exploration and colonisation.

## Introduction

One of the most important tasks for physics is to describe natural observations using a minimal set of principles. This approach allows researchers to understand phenomena of impressive complexity over a broad range of lengths and time scales: from the electronic properties of materials up to the motion of planets. A particularly important example is equilibrium thermodynamics, where the properties and transitions between different states of matter have been traced down to the properties of single atoms or molecules. However, it is still unclear whether this reductionist approach can be successfully employed to describe and understand systems that are far from thermodynamic equilibrium^1,2^, which is one of the central challenges for 21st-century physics.

**Fig. 1:**
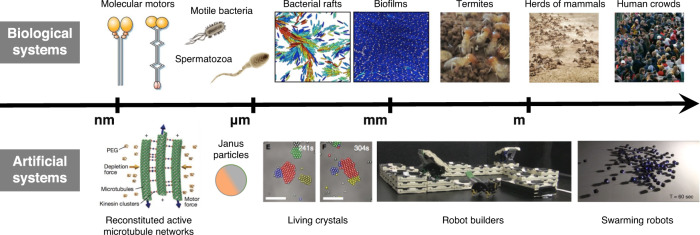
Examples of active matter systems. Overview of active matter systems on all length scales, ranging from nanoscopic molecular and colloidal systems to swarms of animals or robots, and even to human crowds. Despite their seemingly large differences, all these systems exhibit striking similarities regarding their emergent behaviours. Being out of thermodynamic equilibrium, active particles can explore complex forms of dynamical self-organisation and exhibit intricate interplays between single-particle properties and collective behaviours that are impossible at thermodynamic equilibrium. Examples of biological active matter systems (from left to right): biomolecular motors (Reprinted by permission from Springer Nature Customer Service Centre GmbH: Nature, M. Schliwa and G. Woehlke^106^, Copyright 2003 Springer Nature); microorganisms such as motile bacteria and spermatozoa (Reprinted figure with permission from C. Bechinger et al.^3^. Copyright 2016 by the American Physical Society); bacterial rafts and biofilms (Reprinted from H. Jeckel et al.^107^; use permitted by authors of original publication); insects such as termites (From J. Werfel et al.^108^. Reprinted with permission from AAAS); herds of mammals and human crowds (Reprinted from T. Vicsek and A. Zafeiris^6^, Copyright 2012, with permission from Elsevier). Examples of artificial active matter systems (from left to right): reconstituted active microtubule networks (Reprinted by permission from Springer Nature Customer Service Centre GmbH: Nature, T. Sanchez et al.^109^, Copyright 2012 Springer Nature); Janus particles; living crystals (From J. Palacci et al.^110^. Reprinted with permission from AAAS); robotic builders (From J. Werfel et al.^108^. Reprinted with permission from AAAS); swarming robots (Reprinted from Rubenstein et al.^111^, Copyright 2014, with permission from Elsevier).

Active matter provides the ideal tools and materials to seek answers to this tantalising challenge^3–5^. This umbrella term encompasses all living or life-like systems constituted by (a group of) individuals (e.g., microorganisms and other biological entities as well as artificial microscopic particles and robots) that are able to harvest and harness energy and information from their surroundings to generate highly complex and finely synchronised forms of self-organisation^6^. As shown in Fig. 1, examples of these systems are varied and span across length and time scales: molecular motors at the nanoscale^7^; motile biological cells and artificial microswimmers at the microscale^3^; flocking birds, schooling fish, crowds of pedestrians and swarming robots at the macroscale^8^.

Differently from passive matter, whose properties are determined by interaction potentials and action–reaction laws, interactions in active matter (and certainly in living matter) frequently arise from other kinds of forces, which might even dismiss the reciprocity of physical interactions, i.e., for each action there need not be an equal and opposite reaction^9–12^. These forces, which originate from chemical activity, hydrodynamic interactions, and even sensory perception, yield the emergence of collective behaviours and the self-organisation of dynamic structures, materials and devices that can move, morph, cooperate, compete, reproduce, and evolve, thus providing the basic functions of life by autonomously transforming energy^5,7^.

Thanks to their small size and ease of control under standard laboratory conditions, soft active matter systems are particularly apt to shed light on far-from-equilibrium dynamics. These mesoscopic active matter systems are composed of self-propelling microscopic units that can consume available energy to perform tasks autonomously, and share common defining features with other soft matter systems, namely the capability of deforming under the action of small perturbations comparable with thermal energy (*k*_B_*T*, with *k*_B_ the Boltzmann constant and *T* the absolute temperature)^13,14^.

On Earth, the ubiquity of gravity weighs on these soft active matter systems, often eclipsing other contributions and interactions in the vertical direction. In fact, the properties and behaviours of many soft matter systems (including active matter) are often dominated by the presence of gravity, mainly through convection and sedimentation^15,16^. For example, for active particles in liquid environments, gravitation-driven sedimentation limits experimental studies to quasi-two-dimensional geometries or to stressed three-dimensional systems strongly affected by density gradients^15,16^. Density matching then becomes the (difficult and imperfect) method of choice to observe phenomena that would otherwise be concealed or crashed by gravity^15,16^. Nevertheless, gravity is not always a nuisance, but it can also be exploited by active systems to navigate their environment, e.g., by exhibiting gravitactic behaviours^17–19^.

Beyond its fundamental interest as a model system for far-from-equilibrium physics and living matter, active matter has also attracted attention for its potential to develop novel applications for targeted delivery, environmental monitoring and additive manufacturing^20–23^. Soft active materials are already at the heart of highly diverse technological applications on Earth (e.g., in materials science, autonomous agents, enhanced bioremediation and drug delivery) and will play a key role as functional materials enabling the future of space exploration and colonisation, e.g., to develop bioreactors for chemical synthesis and energy conversion in space flight. The use, processing and manufacturing of these materials on Earth and in space are expected to differ significantly because of gravitational phenomena, so a deep level of understanding and control of such processes must be developed in both environments independently. For example, gravity alters the supply of nutrients and waste removal by convective flows in bacterial biofilms, which in turn can alter gene expression and function^24–26^. The absence of gravity can then impact both the efficiency of bioreactors and the effectiveness of antibiotics in space flight.

In the last two decades, significant instrumentation development by ESA and NASA (e.g., in advanced light microscopy on the International Space Station (ISS)^27^) has enabled studies, e.g., of colloidal soft matter^28^, granular matter^29,30^ and complex plasma^31^ in microgravity. Nonetheless, active matter, being a relatively new field of research, is still not well explored in low-gravity and microgravity conditions.

This review surveys the current research in active matter with an emphasis on soft systems that are affected by gravity-driven phenomena. It also provides an outlook on key fundamental knowledge gaps that can benefit from the space programme: Studies in microgravity can indeed help tackle fundamental questions and build a unified understanding of active matter systems and, more generally, of non-equilibrium phenomena also on Earth, while studies at both low gravity (e.g., on the Moon or on Mars) and microgravity (e.g., on the ISS) conditions can provide insight on the development of technologies based on active matter for the future of space exploration and colonisation. Finally, this review evaluates the technical feasibility of tackling these questions based on the current and planned instrumentation available to space programmes.

## Active matter systems

Currently, the approaches used to study active matter depend both on the size and the nature of the system under observation (Fig. 1). In terms of size, we can distinguish between two extreme regimes: microscopic and macroscopic active matter.

*Microscopic* active matter systems include, e.g., active molecules^32^, small microorganisms (such as bacteria^33^ and sperm cells^34^), artificial self-propelling particles^3^, and active nematics^35^ (typically with characteristic dimensions of a few micrometres or less). They are dominated by interaction energies of magnitude comparable to the thermal energy *k*_B_*T* and by the prevalence of viscous forces over inertial effects, as they are often immersed in a liquid environment^36^. This entails a few points worth consideration: (1) the speeds of the active agents are proportional to the instantaneous applied force, as inertia can be safely neglected^36^; (2) Brownian motion and randomness play a major role in determining the trajectories and behaviours of these systems, due to typical energy landscapes comparable to thermal energy^3^; and (3) the interactions between agents are often determined by direct contact and short-range chemical gradients^3^ (even though in some – especially artificial-systems, long-range forces are also possible, e.g., originating from electrostatic interactions and light pressure^37^).

*Macroscopic* active matter systems include e.g, motile animals^6^ and robots^38^ (typically with characteristic dimensions of a fraction of a millimetre or more). Their behaviours are dominated by deterministic and inertial forces as well as by sensory interactions (e.g., enabled by their senses and perception). Thus, they are characterised by relatively large agents that can have very considerable internal complexity^39^. As these agents are not limited by noise, information processing can take place in more complex ways than in microscopic active matter systems^40^. Hence, these larger particles have a clearer agenda, being able to choose what to do in the short, mid and long term^41^. This capability of abstraction, along with more complex forms of interactions based on sensory inputs and internal data processing, can also lead to more complex forms of organisation (e.g., human crowds^42^ and robotic swarms^38^) with respect to microscopic active matter.

This distinction between microscopic and macroscopic active matter systems can be formalised using two dimensionless numbers: the Reynolds number and the Péclet number. The Reynolds number is the ratio between inertial and viscous forces acting on an active particle in a fluid. For example, for a particle of characteristic length *a* moving with speed *v* in a fluid with density *ρ*_f_ and viscosity *η*_f_, the Reynolds number is

1 $${{{\rm{Re}}}}=\frac{{\rho }_{{{{\rm{f}}}}}av}{{\eta }_{{{{\rm{f}}}}}}.$$

${{{\rm{Re}}}}$ is small for microscopic active matter (i.e., ${{{\rm{Re}}}}\ll 1$), and large for macroscopic active matter (i.e., ${{{\rm{Re}}}}\gg 100$). The Péclet number instead characterises the relative importance of directed motion versus diffusion for an active particle, and it is written as

2 $${{{\rm{Pe}}}}=\frac{va}{D},$$

where *D* is the particle’s translational diffusion coefficient. The higher the Péclet number, the less relevant is Brownian motion in determining the dynamics of an active particle.

Active matter systems can also be classified as *living* or *artificial*. Often, the inspiration for the realisation of novel artificial active matter systems comes from living systems that have evolved naturally over billions of years to harness available energy to perform work for different tasks that improve their fitness^43^.

Living microscopic active matter systems include, e.g., bacterial cells^33^, phytoplankton^44^ and sperm cells^34^, while self-propelling colloids are a paradigmatic example of artificial microscopic active matter^3^. One of the most popular realisations of active colloids is represented by Janus particles. These are dielectric particles that can self-propel thanks to a hemispherical coating of a different material (e.g., a metal) with catalytic and hydrodynamics effects^45^ or light-absorbing properties^46,47^. Similar to their living counterparts, micron-sized active colloidal particles are able to convert energy from their surroundings into directed motion and self-organise in collective states such as swarms^48^, flocks^49^, and even swirls^50^. So far, however, the behaviours observed in such artificial systems are rather simple and governed by mere physical forces such as steric, phoretic and hydrodynamic interactions^51,52^. Only recently, more complex behaviours that entail feedback interactions among the units have started to be implemented^53–55^. Moreover, differently, from their living counterparts, which can move by body deformation, most synthetic active particles have rigid shapes with only a few examples deviating from this pattern^56–58^.

Macroscopic examples of living active systems include animal groups and human crowds^6^. Active granular matter^5^ and robotic swarms^38^ are instead examples of artificial macroscopic active systems. By taking advantage of the possibility of programming complex interaction rules, macroscopic robotics provides tools to develop and implement complex control strategies into real systems, well beyond what can be currently realised with simple physical and chemical interactions at the microscopic scale^59^. This approach is particularly alluring to address the “reality gap”, i.e., the questions related to how a complex strategy designed in silico would then play out in a real system^60^. Indeed, as opposed to living systems, where interaction rules are often not precisely known (or measurable), this approach can help study how complex behaviours emerge and change upon systematic variations of precisely known interaction rules with increasing levels of complexity.

## Active matter and gravity

**Fig. 2:**
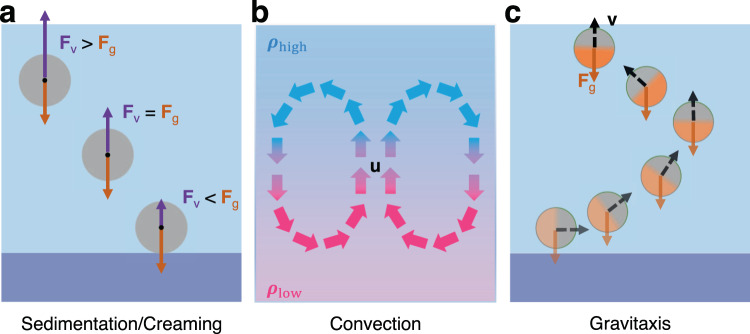
The effects of gravity on active matter. On Earth, microscopic active matter is affected by gravity in multiple ways. The most relevant ones include: **a** Sedimentation and creaming, where the balance between the particle’s and solvent’s densities lead to either an upward or downward motion of the particle in a fluid. *F*_v_ and *F*_g_ represent the viscous force and gravitational force, respectively. **b** Convection, where flow patterns at speed **u** due to density mismatches of various origin transport matter within the sample. The colour scale represents a gradient of density *ρ* going from low density (*ρ*_low_) at the bottom to high density (*ρ*_high_) at the top. **c** Gravitaxis, where a motile organism or synthetic particle with self-propulsion speed **v** moves in a direction influenced by gravitational fields.

On Earth, gravity plays a crucial role in determining the properties and behaviours of active matter systems, particularly at the microscopic scale. Gravity mainly manifests itself because of density heterogeneity either in the active units forming the active matter system or in the solvent where they move. This leads to several phenomena permeating essentially all active matter experiments on Earth. As shown in Fig. 2, the most important ones are: sedimentation (and creaming), convection, and gravitaxis. It is important to note that these phenomena have also influenced the evolution of biological active matter on Earth^61^. In fact, to counteract gravity, living organisms have developed structures, e.g., to regulate fluid flows^62^ and to provide cell membrane rigidity and appropriate structural support for locomotion^63,64^.

### Sedimentation and creaming

As shown in Fig. 2a, under a gravitational field, the interplay between the density of an active particle and the density of the solvent where it is immersed can lead to either sedimentation (when the particle’s density is greater than the density of the solvent) or creaming (when the particle’s density is smaller than the density of the solvent). For example, for a sphere of radius *R* and density *ρ* in a fluid of density *ρ*_f_ and viscosity *η*_f_ (Fig. 2a), the balance between viscous force *F*_v_ = 6*πη*_f_*R* and gravitational force ${F}_{{{{\rm{g}}}}}=\frac{4\pi {R}^{3}}{3}(\rho -{\rho }_{{{{\rm{f}}}}})g$ leads to the sedimentation (*ρ* > *ρ*_f_) or creaming (*ρ* < *ρ*_f_) speed given by

3 $${{{\bf{v}}}}=\frac{2}{9\eta }(\rho -{\rho }_{{{{\rm{f}}}}}){R}^{2}g{{{{\bf{u}}}}}_{z},$$

where *g* is the gravitational acceleration and **u**_*z*_ is the unit vector in the vertical direction. The relative contribution of gravitational and viscous forces can be captured by the Archimedes number:

4 $${{{\rm{Ar}}}}=\frac{g{R}^{3}(\rho -{\rho }_{{{{\rm{f}}}}})}{{\rho }_{{{{\rm{f}}}}}{\eta }_{{{{\rm{f}}}}}^{2}}.$$

Ar is large (i.e., Ar ≫ 1) when the gravitational contribution is dominant over the viscous forces. In the absence of gravity, Ar = 0 and the sedimentation (or creaming) velocity is null.

These gravitational effects can be useful on Earth by aiding multiple units to accumulate at interfaces or surfaces^65^. For example, this can be advantageous to increase encounter rates^66^, e.g., between individuals of the same species, as in the case of biofilm formation^67,68^. Some species of phytoplankton can even adjust their buoyancy in seawater by controlling the rate of aggregation or of dissociation of cytoplasmatic liquid droplets^69^. Furthermore, sedimentation and creaming of nano- and microscopic particles in a solution can give rise to concentration gradients, which can then be exploited by the active particles to perform chemotaxis and diffusiophoresis^70^.

However, the ubiquitous presence of sedimentation and creaming poses some strict limitations for active matter experiments performed on Earth. In particular, it limits the dimensionality of the problems that can be studied^71^: as it induces the localisation of active matter near surfaces and interfaces, most experiments must be conducted in quasi-two-dimensional settings. Moreover, active particles with an intrinsic density heterogeneity such as Janus particles^45,47^ tend to sediment with a preferential orientation determined by a torque produced by the heterogeneous distribution of the particle’s mass, as observed in recent experiments^72,73^.

These limitations can be counteracted with some clever experimental designs, such as by density-matching the solvent^74^, as also done for passive colloids^75^. Nonetheless, this also introduces significant limitations to the accessible experimental systems by, e.g., limiting the range of solvents and particles that can be combined and studied together. Furthermore, when studying phase-separation phenomena, this leads to unwanted sinking or buoyancy of the separated phase. Density matching of living active matter (such as bacteria) presents additional challenges, because cells can adapt to the medium change, in contrast to artificial passive and active particles.

Another alternative experimental design to obviate the effects of gravity is to levitate the systems under study with, e.g., optical or acoustic forces^76^. Yet, this approach imposes additional stresses on the particles under study, and only works for objects of limited size (up to a few mm). For example, when studying the development of an organoid levitated in an acoustic trap, the organoid tends to eventually fall out of the trap because of its own growing weight^77^.

### Convection

As shown in Fig. 2b, convective (or advective) flow patterns arise in the presence of, e.g., temperature, concentration or mass gradients because these induce density mismatches across the sample. For example, convective flows have been reported in phototactic algal systems where uneven mass distribution of cells swimming towards light creates bioconvective plumes leading to cell recirculation^78^. Convective flows in thermal vents have also been suggested to have played a role in the origin of the molecules of life^79^.

Indeed, even in the absence of externally forced flows, a buoyancy-driven flow may still arise because of density gradients in the presence of gravitational forces. This effect is referred to as natural convection, free convection, or simply buoyant convection. In the case where density variations are caused by temperature variations, convection currents can persist dynamically, by contributing to heat and mass transport. The Rayleigh number is a dimensionless number that can be used to express the strength of these temperature-induced buoyancy-driven flows. It is defined as the ratio between the time scale for diffusive thermal transport and the time scale for convective thermal transport at speed *u*:

5 $${{{\rm{Ra}}}}=\frac{{L}^{2}/\alpha }{{\eta }_{{{{\rm{f}}}}}/(\Delta \rho Lg)},$$

where *L* is a characteristic length scale, *α* is the thermal diffusivity, and Δ*ρ* is a characteristic density difference (Δ*ρ* = *ρ*_f_*β*Δ*T* for a fluid of average mass density *ρ*_f_, thermal expansion coefficient *β*, at a temperature difference Δ*T* across the characteristic distance *L*). For the onset of convection, the Rayleigh number has to exceed a threshold value when heating from below a pure fluid. This threshold depends on the boundary conditions (Ra = 1708 for purely conductive boundaries and Ra = 720 for purely impermeable ones).

As for the case of sedimentation and creaming, also uncontrolled convective flows (as those generated by temperature gradients) are a significant limiting factor for active and soft matter experiments on Earth because often these flows have an overwhelming effect on the experiments by overshadowing more subtle phenomena and inducing a (sometimes undesirable) mixing in the solution^80^.

The presence of convective flows is often difficult to counteract. For example, temperature-driven convection in water (a common solvent for active matter experiments) is almost unavoidable due to the relatively large thermal expansion coefficient of water. One way to get around this limitation is by realising sample cells that are thin enough, as the Rayleigh number sharply decreases with the sample cell’s height (*L* in Eq. (5)), but this requires the use of very thin sample cells on the order of a few tens of micrometres to push Ra below the threshold value for the onset of convection (see, e.g.,^81^). Thin samples are not always viable in experiments as they limit the observable phenomena to a quasi-two-dimensional system^71^. Moreover, if the temperature gradient has a component that is perpendicular to the gravitational field, the corresponding thermal convection has no threshold. In the case of thermal convection, another approach to minimise its effects is to ensure that the hotter parts of the solution are at the top to prevent them from moving, but this is often a technical challenge that might limit the range of experiments that can be performed. Furthermore, this option only works for pure fluids and for fluids with solutes characterised by a positive Soret coefficient. For fluids with solutes of negative Soret coefficient, heating from above is enough to avoid thermal convection but is not enough to avoid solutal convection in the presence of gravity^82^.

Finally, it is worth mentioning that, even when convection is under control, the presence of gravity can influence microscopic transport and relaxation. For example, an interesting phenomenon is represented by the non-equilibrium temperature and/or concentration fluctuations that arise as a consequence of the coupling of equilibrium velocity fluctuations with a macroscopic gradient in these quantities^83–85^. On Earth, the amplitude and dynamics of these fluctuations are strongly affected by gravity, which prevents them from growing beyond a certain size and from lasting longer than a limited lapse of time (typically subseconds). In microgravity, they become giant (i.e., with a size that is as large as the container in which they develop) and relax extremely slowly (i.e., with lifetimes of tens of hours)^83–85^. This can have major consequences on various processes in space such as protein crystallisation and phase separations.

### Gravitaxis

As shown in Fig. 2c, gravitaxis (or geotaxis) is characterised by the movement of an organism or synthetic particle in response to gravitational forces^17^. In fact, various swimming microorganisms are able to recognise the alignment of the gravitational field and move accordingly. There are a few different mechanisms for gravitaxis^17^. Many microorganisms have balance receptors (e.g., statocysts) to sense the gravity direction and to adjust their orientation as a consequence. However, gravitaxis can also result from purely physical mechanisms so specialised receptors for sensing the direction of gravity are not always necessary. An example is given by microorganisms with a centre of mass that is shifted to one of their ends. Similar to a buoy, such mass-anisotropic microorganisms orient upwards under gravity^86^. It has been shown that even a slight asymmetry in the shape of microorganisms can be sufficient to cause gravitaxis^87,88^. Indeed, gravity tends to keep the centre of mass at the lowest point possible (a buoy) while activity combined with an anisotropic particle shape leads to an opposing torque (over)compensating the former one.

Some of these physical mechanisms have also been recently reproduced in artificial systems^18,19,89^. In fact, most artificial active particles propel thanks to an asymmetry in their physical and/or chemical properties. As seen in the previous section, Janus colloids constitute a widespread class of active particles. Their asymmetry in materials’ density allows them to show gravitactic behaviours in gravitational fields. In particular, for Janus particles with a heterogeneous mass distribution, there is a gravitational torque^72^, which, in contrast to passive colloidal spheres, cannot be avoided on Earth by density-matching. The presence of this torque on Earth can make it very difficult to separate motility-induced phenomena in active particles from the effects purely due to gravitaxis.

## Outlook for space exploration

As seen in the previous section, many microscopic active matter systems feature an intrinsic density anisotropy both at the individual-particle level (e.g., Janus particles^45,47^) and in mixtures (e.g., active baths^81,90,91^). Because of this density mismatch, many studies conducted on Earth have been limited to quasi-two-dimensional observations due to the difficulty of properly density-matching these systems, which are thus heavily influenced by sedimentation and convection.

By permitting researchers to decouple the effects of gravity, introduce a controllable amount of gravitational effects, and limit the influence of sedimentation and convection, studies in microgravity will provide fundamental insights into the physics of active matter in three dimensions and will also prove useful to develop technological applications in the space environment, such as energy harvesting by light absorption in, e.g., algal fuel cells, as well as microbial bioreactors for CO_2_ removal, hydrogen production, additive materials manufacturing, or bacterial synthesis^92^.

While some scientific questions for microgravity research are shared with other areas of soft matter and have therefore already been explored to some extent (e.g., deformation of soft particles, resistance to harsh conditions, phase transitions and stability^15,16^), there are still several knowledge gaps and priorities for space research that are of specific interest for active matter. Here, we highlight the main ones:

- *Role of gravitational forces and torques on active motion*. On Earth, gravitational forces and torques influence both the translational and rotational degrees of motion of active particles due to density heterogeneity within the particles and with the solvent. Their absence could influence and fundamentally change not only the motion at the single-particle’s level but also the possible emergence of collective behaviours when moving in three dimensions. Studies in space can then provide insights into the effects due to gravity and permit one to distinguish them from the effects due to other factors and interactions, such as due to hydrodynamics for bacterial cells moving near interfaces^68,93^. These forces and torques might also influence the formation of multiscale active biological structures such as microtubules, chromatin, or neurofilaments, which have not yet been achieved in synthetic systems. One of the experimental constraints is that gravitational sedimentation or creaming limits them to nanosize objects. However, in microgravity, one can potentially make much larger entities.
- *Role of weaker forces and interactions*. The absence of gravity will also permit to explore other, weaker forces, such as fluctuation-induced Casimir forces^94^ and their many-body effects^95^, which are typically overshadowed by gravitational effects. These forces can play a major role in the formation of active matter in space, with potential interesting applications. For example, it might be possible to realise some large, three-dimensional active colloidal molecules^11^, which could be useful to realise functional materials based on active matter^73^.
- *Inertial effects in active matter*. Whilst most microscopic active matter exists in an over-damped regime, both translational and rotational inertial effects in active matter become increasingly important for systems where damping is intrinsically low, such as for dusty plasmas and active granular materials^96^. In three-dimensional systems, these effects are overwhelming perturbed by gravity on Earth. In this low-damping regime relevant for inertial particles, density matching no longer works to compensate for gravity. Microgravity is thus needed to isolate the influence of inertia in the motion of individual particles and in the emergence of collective behaviours (e.g., three-dimensional motility-induced phase separations) for low-damped active matter.
- *Role of convection and bioconvection in active matter*. The motility of active systems, such as microorganisms, is also often determined by a complex interaction with their environment. In particular, individual responses can couple to form complex patterns due to density gradients when in Earth’s gravitational field, e.g., due to convective flow patterns. The bioconvective flows observed in phototactic algal systems are a typical example of such complex patterns^78^. To what extent and how these complex patterns can form in the absence of convection is an open question.
- *Emerging behaviours in three-dimensional mixtures of active and passive particles and active baths*. Already in two dimensions, a lot of emerging behaviours have been observed in mixtures of active and passive colloids, including sorting and segregation^81^, cargo transport^97,98^ and material nucleation^99^. In general, such collective phenomena in similar mixtures can be influenced by density heterogeneity, especially when moving to three-dimensional settings, due to, e.g., sedimentation. It is therefore a question of fundamental interest to understand the role of density mismatches in determining such collective properties. For example, the dimensions and scales of aggregates have been shown to increase in soft matter systems in microgravity^83,100^, so similar phenomena can also be expected to emerge for active particles in microgravity.
- *Aggregation and self-assembly*. In terrestrial environments, the size of aggregates of active matter in bulk is limited by the presence of gravitational forces. This for example limits the size of active materials that can be assembled or of organoids that can be grown far from the coverslip surface^77^. Cell adhesion and cell–cell interactions are also heavily affected by gravity impairing the formation and extension of multicellular aggregates^101,102^. These limitations can be overcome in microgravity, providing new insights, e.g., on the collective organisation of active particles, on tissue growth and morphogenesis. Moreover, the emergence of collective phenomena that are qualitatively different in 2D and 3D could be studied. Finally, very different levels of connectivity are achievable in 2D and 3D geometries, which has consequences, e.g., for the growth of neuronal networks as well as for the possible application of active matter to create self-organised neuromorphic computers^59^.
- *Radiation effects on active matter*. The space environment is characterised by high-radiation conditions. Understanding how these influence the activity, physical properties and durability of materials is important to ensure the use of active matter systems in space exploration and colonisation. For example, for biological active matter, high levels of radiation can indeed inflict DNA damage and mutation that can determine a change in both individual and collective behaviours, thus affecting the performance of active materials based on such biological structures. This understanding will be essential in light of the future installation of permanent bases on the Moon and, later, on Mars.
- *Active matter in extreme environments*. These include research into the effects of ultra-high vacuum, extreme thermal gradients and extreme radiation effects. All these phenomena can have a significant impact, especially on living active matter, such as bacterial cells exposed to different environmental conditions, which might be relevant for their use as bioreactors in space, e.g., to synthesise drugs and chemicals, remove CO_2_, or produce hydrogen.

## Experimental design for microgravity research

**Fig. 3:**
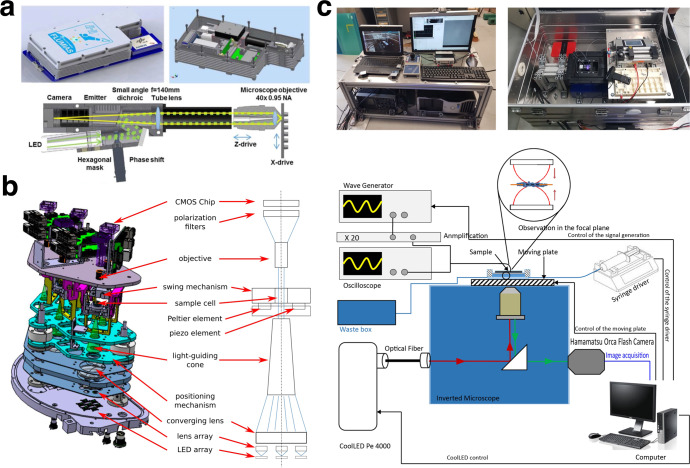
Examples of optical microscopy setups for microgravity research. **a** FLUMIAS is a miniaturised high-resolution 3D fluorescence microscope coupled to a centrifuge on the International Space Station (ISS) for live cell imaging. FLUMIAS can therefore be operated in microgravity as well as in controlled artificial gravity conditions with an effective gravitational acceleration of up to 1.1×*g*. Reprinted from C. S. Thiel et al.^27^; use permitted by authors of original publication. **b** The RAMSES flight platform is designed for the sounding rocket MAPHEUS to study the motion of self-propelled particles in space. Reprinted from R. Keßler et al.^71^, with the permission of AIP Publishing. **c** Optical microscopy setup used on the Airbus Zero-G for the acoustic manipulation of dense gold nanorods samples in microgravity. Reprinted by permission from Springer Nature Customer Service Centre GmbH: Springer, G. Dumy et al.^76^, Copyright 2020 Springer Nature.

Several of the challenges listed above can be addressed by fitting new samples and sample cells into existing instruments on the ISS or by employing experimental devices that are already under development for microgravity research, but have not made it to the ISS yet. In fact, both novel hardware designs and techniques have recently been proposed for running soft matter experiments in microgravity conditions with versatile platforms that could also be adapted for active matter experiments in a straightforward way^103^. For example, FLUMIAS (FLUorescence MIcroscopy Analysis System) is a miniaturised fluorescence microscopy technology coupled to a centrifuge which has been proposed to study fixed and live cells on the ISS^27^ and could be adapted to studies of microscopic biological active matter (Fig. 3a)^27^. As another example, RAMSES (RAndom Motion of SElf-propelled particles in Space) is a highly integrated automated experimental module that has been designed to study the active Brownian motion of light-driven colloidal Janus particles on the sounding rocket platform MAPHEUS (MAterialPHysikalische Experimente Unter Schwerelosigkeit) (Fig. 3b)^71^. These platforms could also be enhanced by introducing further functionalities via thermal control capabilities^80^ or fields, e.g., for optical and acoustic manipulation^76^ (Fig. 3c).

**Fig. 4:**
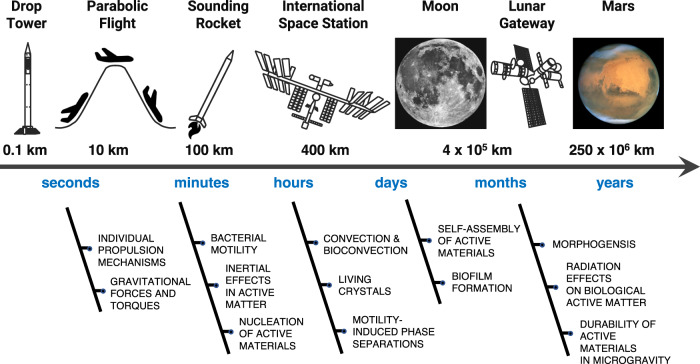
The future of space exploration for active matter. In the near future (<3 years), we can expect an increase in active matter experiments on the International Space Station (ISS) in low Earth orbit (LEO) based on using or adapting existing instrumentation for soft matter studies in microgravity. The main advantage of running active matter experiments on the ISS is the possibility of studying these systems under microgravity conditions for up to several hours. In the mid (5–10 years) and long term (>10 years), fitting this instrumentation on Moon, Mars and Beyond-Low-Earth-Orbit (BLEO) missions (e.g., on the Lunar Gateway) will enable testing active matter for the future of space exploration and colonisation on even longer time scales (up to a few years or longer). Moon picture credit: ESA/Hubble; use permitted under a CC-BY 4.0 licence. Mars picture credit: ESA/Hubble; use permitted under a CC-BY 4.0 licence.

The main advantage of running active matter experiments on the ISS in low Earth orbit (LEO) is the possibility to extend them to at least several hours (Fig. 4). In fact, while different optical microscopy platforms exist for microgravity studies of soft matter systems on Earth that can be adapted to study active matter, these are typically limited by the time scales that can be realistically accessed: experiments on drop towers and parabolic flights can reproduce microgravity conditions only for times up to 9 s and 22 s, respectively; experiments on sounding rockets can last up to 12 mins.

Beyond existing instrumentation, the development of novel instrumentation on the ISS will also speed up the discovery process of novel phenomena in soft matter and active matter under microgravity conditions. Furthermore, fitting this instrumentation on Moon, Mars, and Beyond-Low-Earth-Orbit (BLEO) missions will enable testing active matter for future space exploration and colonisation on even longer time scales (up to a few years or longer).

Moving forward, it is hence important to ensure that experiments in microgravity can be run in parallel and in an automated way to enable high-throughput sample handling and control. To this end, miniaturisation is key. Luckily, experiments with microscopic active matter are very apt for this purpose as they typically involve small volumes (<1 μL droplets), can rely on the use of microfluidic lab-on-a-chip designs^59,104^ and are typically observable under an optical microscope. Another key factor will be the inclusion of machine learning approaches in the experimental design and data analysis strategies to deal with a limited amount of materials and information in an optimal way^59^. Finally, the possibility of recycling experimental materials is another alluring feature to incorporate into the experimental design, where limited availability or replacement of experimental resources might limit the viability of experiments in the future of space exploration.

## Future outlook and summary

The possibility of studying active matter in low gravity and microgravity is promising both on a fundamental level and on an applied level. These studies become particularly relevant when moving from systems confined to two dimensions to three-dimensional systems. In particular, the possibility of opening active matter studies to the third dimension with microgravity will enable a much greater variety of individual and collective phenomena, forms of self-organisation and materials to be studied and developed. On the fundamental level, studies in microgravity will enable researchers and engineers to factor out gravitational forces and to isolate the fundamental physical and chemical interactions that determine the emergence of both individual and collective behaviours in synthetic and biological active matter systems. On the applied level, the study of active matter in low-gravity environments (e.g., on the Moon and on Mars) and in space is expected to lead to new technological applications and advanced materials, overall permitting researchers to take control of active matter both in the terrestrial and space environments. For example, the study of artificial active matter will generate a better understanding of the active transport mechanisms existing in biological cells, thus enabling new forms of drug delivery to be developed. Importantly, a greater level of understanding will also be required to devise novel materials and manufacturing processes based on active matter that can efficiently operate in space. Future space exploration will undoubtedly require in situ manufacturing capabilities and a better understanding of material processing can only result by carrying out ad hoc microgravity studies and compare them with similar studies on Earth.

Here we focused on microgravity, yet similar research questions and challenges as those discussed in this review can also be extended to the case of hypergravity^105^. Whilst interesting from a fundamental physics point of view, these studies are less pressing for the more technical aspects connected to the foreseeable future of space exploration.

In summary, despite the fact that other fields in soft matter and beyond have benefited from previous research in space, active matter, due to its young age as a research field, has still not seen a real uptake of studies in microgravity. Nonetheless, while we advance our understanding of far-from-equilibrium physics and statistical mechanics on Earth, the scope for learning more about these scientific challenges of our century by sending active matter into space is broad and still widely untapped.

### Reporting summary

Further information on research design is available in the Nature Research Reporting Summary linked to this article.

## Supplementary information

Reporting Summary

Supplementary Information

## Data Availability

No datasets were analysed or generated in the writing of this review.

## Code availability

No codes were used in the writing of this review.

## References

[1] Goldenfeld N, Woese C (2011). Life is physics: evolution as a collective phenomenon far from equilibrium. Annu. Rev. Condens. Matter Phys..

[2] Fodor É (2016). How far from equilibrium is active matter?. Phys. Rev. Lett..

[3] Bechinger C (2016). Active particles in complex and crowded environments. Rev. Mod. Phys..

[4] Popkin G (2016). The physics of life. Nature.

[5] Ramaswamy S (2017). Active matter. J. Stat. Mech. Theory Exp..

[6] Vicsek T, Zafeiris A (2012). Collective motion. Phys. Rep..

[7] Needleman D, Dogic Z (2017). Active matter at the interface between materials science and cell biology. Nat. Rev. Mater..

[8] Moussaid M, Garnier S, Theraulaz G, Helbing D (2009). Collective information processing and pattern formation in swarms, flocks, and crowds. Top. Cogn. Sci..

[9] Soto R, Golestanian R (2014). Self-assembly of catalytically active colloidal molecules: tailoring activity through surface chemistry. Phys. Rev. Lett..

[10] Ivlev AV (2015). Statistical mechanics where Newton?s third law is broken. Phys. Rev. X.

[11] Schmidt F, Liebchen B, Löwen H, Volpe G (2019). Light-controlled assembly of active colloidal molecules. J. Chem. Phys..

[12] Das M, Schmidt CF, Murrell M (2020). Introduction to active matter. Soft Matter.

[13] De Gennes P-G (1992). Soft matter. Science.

[14] Doi, M. Soft matter physics (Oxford University Press, 2013).

[15] ESA. ESA SciSpacE white papers: soft matter and biophysics, https://esamultimedia.esa.int/docs/hre/04_physical_sciences_soft-matter-biophysics.pdf (2021).

[16] Chaikin, P., Clark, N. & Nagel, S. Grand challenges in soft matter science: prospects for microgravity research. *Tech. Rep*. (2021).

[17] Häder D-P, Lebert M, Richter P, Ntefidou M (2003). Gravitaxis and graviperception in flagellates. Adv. Space Res..

[18] Campbell AI, Ebbens SJ (2013). Gravitaxis in spherical Janus swimming devices. Langmuir.

[19] ten Hagen B (2014). Gravitaxis of asymmetric self-propelled colloidal particles. Nat. Commun..

[20] Bayíndír L (2016). A review of swarm robotics tasks. Neurocomputing.

[21] Luo M, Feng Y, Wang T, Guan J (2018). Micro-/nanorobots at work in active drug delivery. Adv. Funct. Mater..

[22] Mallory SA, Valeriani C, Cacciuto A (2018). An active approach to colloidal self-assembly. Annu. Rev. Phys. Chem..

[23] Ghosh A, Xu W, Gupta N, Gracias DH (2020). Active matter therapeutics. Nano Today.

[24] Klaus D, Simske S, Todd P, Stodieck L (1997). Investigation of space flight effects on escherichia coli and a proposed model of underlying physical mechanisms. Microbiology.

[25] Kim W (2013). Spaceflight promotes biofilm formation by pseudomonas aeruginosa. PloS One.

[26] Acres JM, Youngapelian MJ, Nadeau J (2021). The influence of spaceflight and simulated microgravity on bacterial motility and chemotaxis. npj Microgravity.

[27] Thiel CS (2019). Real-time 3d high-resolution microscopy of human cells on the international space station. Int. J. Mol. Sci..

[28] Zhu J (1997). Crystallization of hard-sphere colloids in microgravity. Nature.

[29] Bérut A (2018). Gravisensors in plant cells behave like an active granular liquid. Proc. Natl Acad. Sci. USA.

[30] Pitikaris S, Bartz P, Yu P, Cristoforetti S, Sperl M (2022). Granular cooling of ellipsoidal particles in microgravity. npj Microgravity.

[31] Morfill GE, Ivlev AV (2009). Complex plasmas: an interdisciplinary research field. Rev. Mod. Phys..

[32] Kassem S (2017). Artificial molecular motors. Chem. Soc. Rev..

[33] Wadhwa N, Berg HC (2021). Bacterial motility: machinery and mechanisms. Nat. Rev. Microbiol..

[34] Gaffney EA, Gadêlha H, Smith DJ, Blake JR, Kirkman-Brown JC (2011). Mammalian sperm motility: observation and theory. Annu. Rev. Fluid Mech..

[35] Doostmohammadi A, Ignés-Mullol J, Yeomans JM, Sagués F (2018). Active nematics. Nat. Commun..

[36] Purcell EM (1977). Life at low Reynolds number. Am. J. Phys..

[37] Dietrich K (2018). Active atoms and interstitials in two-dimensional colloidal crystals. Phys. Rev. Lett..

[38] Dorigo, M., Theraulaz, G. & Trianni, V. Reflections on the future of swarm robotics. *Sci. Robot*. **5**, eabe4385 (2020). 10.1126/scirobotics.abe4385 33298518

[39] Doncieux S, Bredeche N, Mouret J-B, Eiben AE (2015). Evolutionary robotics: what, why, and where to. Front. Robot. AI.

[40] Gardi G, Ceron S, Wang W, Petersen K, Sitti M (2022). Microrobot collectives with reconfigurable morphologies, behaviors, and functions. Nat. Commun..

[41] Rubenstein M, Cornejo A, Nagpal R (2014). Programmable self-assembly in a thousand-robot swarm. Science.

[42] Bain N, Bartolo D (2019). Dynamic response and hydrodynamics of polarized crowds. Science.

[43] England JL (2015). Dissipative adaptation in driven self-assembly. Nat. Nanotech..

[44] Sengupta A, Carrara F, Stocker R (2017). Phytoplankton can actively diversify their migration strategy in response to turbulent cues. Nature.

[45] Howse JR (2007). Self-motile colloidal particles: from directed propulsion to random walk. Phys. Rev. Lett..

[46] Jiang H-R, Yoshinaga N, Sano M (2010). Active motion of a Janus particle by self-thermophoresis in a defocused laser beam. Phys. Rev. Lett..

[47] Buttinoni I, Volpe G, Kümmel F, Volpe G, Bechinger C (2012). Active Brownian motion tunable by light. J. Phys. Condens. Matter.

[48] Kokot G, Snezhko A (2018). Manipulation of emergent vortices in swarms of magnetic rollers. Nat. Commun..

[49] Bricard A, Caussin J-B, Desreumaux N, Dauchot O, Bartolo D (2013). Emergence of macroscopic directed motion in populations of motile colloids. Nature.

[50] Aubret A, Youssef M, Sacanna S, Palacci J (2018). Targeted assembly and synchronization of self-spinning microgears. Nat. Phys..

[51] Ginot F, Theurkauff I, Detcheverry F, Ybert C, Cottin-Bizonne C (2018). Aggregation-fragmentation and individual dynamics of active clusters. Nat. Commun..

[52] Stark H (2018). Artificial chemotaxis of self-phoretic active colloids: collective behavior. Acc. Chem. Res..

[53] Khadka U, Holubec V, Yang H, Cichos F (2018). Active particles bound by information flows. Nat. Commun..

[54] Lavergne FA, Wendehenne H, Bäuerle T, Bechinger C (2019). Group formation and cohesion of active particles with visual perception–dependent motility. Science.

[55] Muiños-Landin S, Fischer A, Holubec V, Cichos F (2021). Reinforcement learning with artificial microswimmers. Sci. Robot..

[56] Dreyfus R (2005). Microscopic artificial swimmers. Nature.

[57] Palagi S (2016). Structured light enables biomimetic swimming and versatile locomotion of photoresponsive soft microrobots. Nat. Mater..

[58] Alvarez L (2021). Reconfigurable artificial microswimmers with internal feedback. Nat. Commun..

[59] Cichos F, Gustavsson K, Mehlig B, Volpe G (2020). Machine learning for active matter. Nat. Mach. Intell..

[60] Koos, S., Mouret, J.-B. & Doncieux, S. Crossing the reality gap in evolutionary robotics by promoting transferable controllers. In *Proc. 2019 Genet. Evol. Comput. Conf*., 119–126 (2010).

[61] Morey-Holton, E. R. Evolution on Planet Earth. Part 4: gravity, 143–159 (Academic Press, London, 2003).

[62] Adamopoulos K, Koutsouris D, Zaravinos A, Lambrou GI (2021). Gravitational influence on human living systems and the evolution of species on earth. Molecules.

[63] Papaseit C, Pochon N, Tabony J (2000). Microtubule self-organization is gravity-dependent. Proc. Natl Acad. Sci. USA.

[64] Najrana T, Sanchez-Esteban J (2016). Mechanotransduction as an adaptation to gravity. Front. Pediatr..

[65] Kuhr J-T, Blaschke J, Rühle F, Stark H (2017). Collective sedimentation of squirmers under gravity. Soft Matter.

[66] Araújo, N. A. et al. Steering self-organisation through confinement. Preprint at https://arxiv.org/abs/2204.10059 (2022).

[67] van Loosdrecht MC, Lyklema J, Norde W, Zehnder A (1990). Influence of interfaces on microbial activity. Microbiol. Rev..

[68] Makarchuk S, Braz VC, Araújo NA, Ciric L, Volpe G (2019). Enhanced propagation of motile bacteria on surfaces due to forward scattering. Nat. Commun..

[69] Sengupta, A. et al. Active reconfiguration of cytoplasmic lipid droplets governs migration of nutrient-limited phytoplankton. Preprint at https://www.biorxiv.org/content/10.1101/2021.10.17.463831v1 (2021). 10.1126/sciadv.abn6005 PMC11633079 36332020

[70] Palacci J, Cottin-Bizonne C, Ybert C, Bocquet L (2010). Sedimentation and effective temperature of active colloidal suspensions. Phys. Rev. Lett..

[71] Keßler R (2020). Direct-imaging of light-driven colloidal Janus particles in weightlessness. Rev. Sci. Instrum..

[72] Das S (2015). Boundaries can steer active Janus spheres. Nat. Commun..

[73] Trivedi M, Saxena D, Ng WK, Sapienza R, Volpe G (2022). Self-organized lasers from reconfigurable colloidal assemblies. Nat. Phys..

[74] Jeanneret R, Pushkin DO, Kantsler V, Polin M (2016). Entrainment dominates the interaction of microalgae with micron-sized objects. Nat. Commun..

[75] Lu PJ (2008). Gelation of particles with short-range attraction. Nature.

[76] Dumy G (2020). Acoustic manipulation of dense nanorods in microgravity. Microgravity Sci. Technol..

[77] Jeger-Madiot N (2021). Self-organization and culture of mesenchymal stem cell spheroids in acoustic levitation. Sci. Rep..

[78] Arrieta J, Polin M, Saleta-Piersanti R, Tuval I (2019). Light control of localized photobioconvection. Phys. Rev. Lett..

[79] Braun D, Libchaber A (2004). Thermal force approach to molecular evolution. Phys. Biol..

[80] Braibanti M (2019). European space agency experiments on thermodiffusion of fluid mixtures in space. Eur. Phys. J. E.

[81] Pinçe E (2016). Disorder-mediated crowd control in an active matter system. Nat. Commun..

[82] Cerbino R, Vailati A, Giglio M (2002). Soret driven convection in a colloidal solution heated from above at very large solutal rayleigh number. Phys. Rev. E.

[83] Vailati A (2011). Fractal fronts of diffusion in microgravity. Nat. Commun..

[84] Takacs CJ (2011). Thermal fluctuations in a layer of liquid c*s*_2_ subjected to temperature gradients with and without the influence of gravity. Phys. Rev. Lett..

[85] Cerbino R, Sun Y, Donev A, Vailati A (2015). Dynamic scaling for the growth of non-equilibrium fluctuations during thermophoretic diffusion in microgravity. Sci. Rep..

[86] Durham WM, Kessler JO, Stocker R (2009). Disruption of vertical motility by shear triggers formation of thin phytoplankton layers. Science.

[87] Mogami Y, Ishii J, Baba SA (2001). Theoretical and experimental dissection of gravity-dependent mechanical orientation in gravitactic microorganisms. Biol. Bull..

[88] Roberts A (2010). The mechanics of gravitaxis in paramecium. J. Exp. Biol..

[89] Brosseau Q (2021). Metallic microswimmers driven up the wall by gravity. Soft Matter.

[90] Wu X-L, Libchaber A (2000). Particle diffusion in a quasi-two-dimensional bacterial bath. Phys. Rev. Lett..

[91] Di Leonardo R (2010). Bacterial ratchet motors. Proc. Natl Acad. Sci. USA.

[92] Jemison M, Olabisi R (2021). Biomaterials for human space exploration: a review of their untapped potential. Acta Biomater..

[93] Perez Ipiña E, Otte S, Pontier-Bres R, Czerucka D, Peruani F (2019). Bacteria display optimal transport near surfaces. Nat. Phys..

[94] Callegari A, Magazzù A, Gambassi A, Volpe G (2021). Optical trapping and critical casimir forces. Eur. Phys. J..

[95] Paladugu S (2016). Nonadditivity of critical casimir forces. Nat. Commun..

[96] Löwen H (2020). Inertial effects of self-propelled particles: from active Brownian to active Langevin motion. J. Chem. Phys..

[97] Gao W, Pei A, Feng X, Hennessy C, Wang J (2013). Organized self-assembly of Janus micromotors with hydrophobic hemispheres. J. Am. Chem. Soc..

[98] Wang W, Duan W, Sen A, Mallouk TE (2013). Catalytically powered dynamic assembly of rod-shaped nanomotors and passive tracer particles. Proc. Natl Acad. Sci. USA.

[99] Singh DP, Choudhury U, Fischer P, Mark AG (2017). Non-equilibrium assembly of light-activated colloidal mixtures. Adv. Mater..

[100] Veen SJ (2012). Colloidal aggregation in microgravity by critical Casimir forces. Phys. Rev. Lett..

[101] Buravkova L, Romanov Y, Rykova M, Grigorieva O, Merzlikina N (2005). Cell-to-cell interactions in changed gravity: ground-based and flight experiments. Acta Astronaut..

[102] Lin X (2020). The impact of spaceflight and simulated microgravity on cell adhesion. Int. J. Mol. Sci..

[103] Born P (2021). Soft matter dynamics: a versatile microgravity platform to study dynamics in soft matter. Rev. Sci. Instrum..

[104] Sharan P, Nsamela A, Lesher-Pérez SC, Simmchen J (2021). Microfluidics for microswimmers: engineering novel swimmers and constructing swimming lanes on the microscale, a tutorial review. Small.

[105] van Loon JJ (2008). The large diameter centrifuge, ldc, for life and physical sciences and technology. Life Space Life Earth.

[106] Schliwa M, Woehlke G (2003). Molecular motors. Nature.

[107] Jeckel H (2019). Learning the space-time phase diagram of bacterial swarm expansion. Proc. Natl Acad. Sci. USA.

[108] Werfel J, Petersen K, Nagpal R (2014). Designing collective behavior in a termite-inspired robot construction team. Science.

[109] Sanchez T, Chen DT, DeCamp SJ, Heymann M, Dogic Z (2012). Spontaneous motion in hierarchically assembled active matter. Nature.

[110] Palacci J, Sacanna S, Steinberg AP, Pine DJ, Chaikin PM (2013). Living crystals of light-activated colloidal surfers. Science.

[111] Rubenstein M, Ahler C, Hoff N, Cabrera A, Nagpal R (2014). Kilobot: a low cost robot with scalable operations designed for collective behaviors. Rob. Auton. Syst..

