# Publisher Correction: Active matter in space

**DOI:** 10.1038/s41526-023-00263-6

**Published:** 2023-02-17

**Authors:** Giorgio Volpe, Clemens Bechinger, Frank Cichos, Ramin Golestanian, Hartmut Löwen, Matthias Sperl, Giovanni Volpe

**Affiliations:** 1 Department of Chemistry, University College London, 20 Gordon Street, WC1H 0AJ London, United Kingdom; 2 Physics Department, University of Konstanz, 78457 Konstanz, Germany; 3 Peter Debye Institute for Soft Matter Physics, Faculty of Physics and Earth Sciences, Leipzig University, 04103 Leipzig, Germany; 4 Max Planck Institute for Dynamics and Self-Organization (MPI-DS), 37077 Göttingen, Germany; 5 Rudolf Peierls Centre for Theoretical Physics, University of Oxford, Oxford, OX1 3PU United Kingdom; 6 Institut für Theoretische Physik II: Weiche Materie, Heinrich-Heine-Universität Düsseldorf, Universitätsstrasse 1, 40225 Düsseldorf, Germany; 7 Institut für Materialphysik im Weltraum, Deutsches Zentrum für Luft- und Raumfahrt (DLR), 51170 Köln, Germany; 8 Physics Department, University of Gothenburg, 41296 Gothenburg, Sweden

**Keywords:** Structure of solids and liquids, Soft materials, Biophysics, Structure of solids and liquids, Colloids

Correction to: *npj Microgravity* 10.1038/s41526-022-00230-7, published online 24 November 2022

In the original version of the Article, the photo of Mars in Figure 4 has a CC BY-SA 3.0 IGO licence, incompatible with the [CC-BY 4.0] license under which the article has been made available. The permitted use version of Figure 4 is
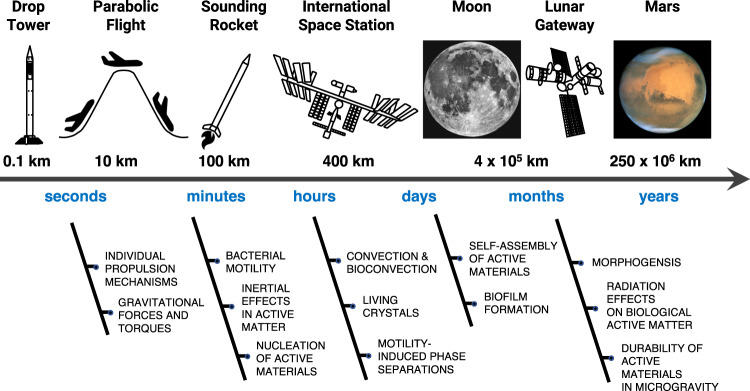


which replaces the previous version.

In association to this error, the legend to Figure inadvertently missed the licence link in the last sentence. The correct version reads as, ‘Moon picture credit: ESA/Hubble; use permitted under a [CC-BY 4.0 licence]. Mars picture credit: ESA/Hubble; use permitted under a [CC-BY 4.0] licence.’

