# Supplementary material for: Active matter in space
Source: NPJ Microgravity. 2022 Nov 24;8:54. doi: 10.1038/s41526-022-00230-7 (PMC9700843; doi:10.1038/s41526-022-00230-7)
Supplement: Supplementary file 2 — Supplementary Information [file 41526_2022_230_MOESM2_ESM.zip › Final Permissions/Figure 1/Fig1_Designing Collective Behavior in a Termite-Inspired Robot Construction Team.pdf]

THE AMERICAN ASSOCIATION FOR THE ADVANCEMENT OF SCIENCE LICENSE TERMS AND CONDITIONS

Apr 28, 2022

This Agreement between Giorgio Volpe ("You") and The American Association for the Advancement of Science ("The American Association for the Advancement of Science") consists of your license details and the terms and conditions provided by The American Association for the Advancement of Science and Copyright Clearance Center.

License Number	5297570501236
License date	Apr 28, 2022
Licensed Content Publisher	The American Association for the Advancement of Science
Licensed Content Publication	Science
Licensed Content Title	Designing Collective Behavior in a Termite-Inspired Robot Construction Team
Licensed Content Author	Justin Werfel, Kirstin Petersen, Radhika Nagpal
Licensed Content Date	Feb 14, 2014
Licensed Content Volume	343
Licensed Content Issue	6172
Volume number	343
Issue number	6172

Type of Use	Journal
Requestor type	Scientist/individual at a research institution
Format	Print and Electronic
Portion	Figure
Number of figures/tables	2
Title of new article	Active Matter in Space
Lead author	Giorgio Volpe
Title of targeted journal	NPJ Microgravity
Publisher	Springer Nature
Expected publication date	Sep 2022
Portions	Fig. 1, panel B; Fig. 4, panel A
Requestor Location	Giorgio Volpe<br>Department of Chemistry, UCL<br>20 Gordon Street<br><br>London, WC1H 0AJ<br>United Kingdom<br>Attn: Giorgio Volpe
Total	0.00 GBP

Terms and Conditions

American Association for the Advancement of Science TERMS AND CONDITIONS

Regarding your request, we are pleased to grant you non-exclusive, non-transferable permission, to republish the AAAS material identified above in your work identified above, subject to the terms and conditions herein. We must be contacted for permission for any uses

other than those specifically identified in your request above.

The following credit line must be printed along with the AAAS material: "From [Full Reference Citation]. Reprinted with permission from AAAS."

All required credit lines and notices must be visible any time a user accesses any part of the AAAS material and must appear on any printed copies and authorized user might make.

This permission does not apply to figures / photos / artwork or any other content or materials included in your work that are credited to non-AAAS sources. If the requested material is sourced to or references non-AAAS sources, you must obtain authorization from that source as well before using that material. You agree to hold harmless and indemnify AAAS against any claims arising from your use of any content in your work that is credited to non-AAAS sources.

If the AAAS material covered by this permission was published in Science during the years 1974 - 1994, you must also obtain permission from the author, who may grant or withhold permission, and who may or may not charge a fee if permission is granted. See original article for author's address. This condition does not apply to news articles.

The AAAS material may not be modified or altered except that figures and tables may be modified with permission from the author. Author permission for any such changes must be secured prior to your use.

Whenever possible, we ask that electronic uses of the AAAS material permitted herein include a hyperlink to the original work on AAAS's website (hyperlink may be embedded in the reference citation).

AAAS material reproduced in your work identified herein must not account for more than 30% of the total contents of that work.

AAAS must publish the full paper prior to use of any text.

AAAS material must not imply any endorsement by the American Association for the Advancement of Science.

This permission is not valid for the use of the AAAS and/or Science logos.

AAAS makes no representations or warranties as to the accuracy of any information contained in the AAAS material covered by this permission, including any warranties of

merchantability or fitness for a particular purpose.

If permission fees for this use are waived, please note that AAAS reserves the right to charge for reproduction of this material in the future.

Permission is not valid unless payment is received within sixty (60) days of the issuance of this permission. If payment is not received within this time period then all rights granted herein shall be revoked and this permission will be considered null and void.

In the event of breach of any of the terms and conditions herein or any of CCC's Billing and Payment terms and conditions, all rights granted herein shall be revoked and this permission will be considered null and void.

AAAS reserves the right to terminate this permission and all rights granted herein at its discretion, for any purpose, at any time. In the event that AAAS elects to terminate this

permission, you will have no further right to publish, publicly perform, publicly display, distribute or otherwise use any matter in which the AAAS content had been included, and all fees paid hereunder shall be fully refunded to you. Notification of termination will be sent to the contact information as supplied by you during the request process and termination shall be immediate upon sending the notice. Neither AAAS nor CCC shall be liable for any costs, expenses, or damages you may incur as a result of the termination of this permission, beyond the refund noted above.

This Permission may not be amended except by written document signed by both parties.

The terms above are applicable to all permissions granted for the use of AAAS material. Below you will find additional conditions that apply to your particular type of use.

FOR A THESIS OR DISSERTATION
If you are using figure(s)/table(s), permission is granted for use in print and electronic versions of your dissertation or thesis. A full text article may be used in print versions only of a dissertation or thesis.

Permission covers the distribution of your dissertation or thesis on demand by ProQuest / UMI, provided the AAAS material covered by this permission remains in situ.

If you are an Original Author on the AAAS article being reproduced, please refer to your License to Publish for rules on reproducing your paper in a dissertation or thesis.

FOR JOURNALS:
Permission covers both print and electronic versions of your journal article, however the AAAS material may not be used in any manner other than within the context of your article.

FOR BOOKS/TEXTBOOKS:
If this license is to reuse figures/tables, then permission is granted for non-exclusive world rights in all languages in both print and electronic formats (electronic formats are defined below).

If this license is to reuse a text excerpt or a full text article, then permission is granted for non-exclusive world rights in English only. You have the option of securing either print or electronic rights or both, but electronic rights are not automatically granted and do garner additional fees. Permission for translations of text excerpts or full text articles into other languages must be obtained separately.

Licenses granted for use of AAAS material in electronic format books/textbooks are valid only in cases where the electronic version is equivalent to or substitutes for the print version of the book/textbook. The AAAS material reproduced as permitted herein must remain in situ and must not be exploited separately (for example, if permission covers the use of a full text article, the article may not be offered for access or for purchase as a stand-alone unit), except in the case of permitted textbook companions as noted below.

You must include the following notice in any electronic versions, either adjacent to the reprinted AAAS material or in the terms and conditions for use of your electronic products: "Readers may view, browse, and/or download material for temporary copying purposes only, provided these uses are for noncommercial personal purposes. Except as provided by law, this material may not be further reproduced, distributed, transmitted, modified, adapted, performed, displayed, published, or sold in whole or in part, without prior written permission from the publisher."

If your book is an academic textbook, permission covers the following companions to your

textbook, provided such companions are distributed only in conjunction with your textbook at no additional cost to the user:

- Password-protected website
- Instructor's image CD/DVD and/or PowerPoint resource
- Student CD/DVD

All companions must contain instructions to users that the AAAS material may be used for non-commercial, classroom purposes only. Any other uses require the prior written permission from AAAS.

If your license is for the use of AAAS Figures/Tables, then the electronic rights granted herein permit use of the Licensed Material in any Custom Databases that you distribute the electronic versions of your textbook through, so long as the Licensed Material remains within the context of a chapter of the title identified in your request and cannot be downloaded by a user as an independent image file.

Rights also extend to copies/files of your Work (as described above) that you are required to provide for use by the visually and/or print disabled in compliance with state and federal laws.

This permission only covers a single edition of your work as identified in your request.

FOR NEWSLETTERS:
Permission covers print and/or electronic versions, provided the AAAS material reproduced as permitted herein remains in situ and is not exploited separately (for example, if permission covers the use of a full text article, the article may not be offered for access or for purchase as a stand-alone unit)

FOR ANNUAL REPORTS:
Permission covers print and electronic versions provided the AAAS material reproduced as permitted herein remains in situ and is not exploited separately (for example, if permission covers the use of a full text article, the article may not be offered for access or for purchase as a stand-alone unit)

FOR PROMOTIONAL/MARKETING USES:
Permission covers the use of AAAS material in promotional or marketing pieces such as information packets, media kits, product slide kits, brochures, or flyers limited to a single print run. The AAAS Material may not be used in any manner which implies endorsement or promotion by the American Association for the Advancement of Science (AAAS) or Science of any product or service. AAAS does not permit the reproduction of its name, logo or text on promotional literature.

If permission to use a full text article is permitted, The Science article covered by this permission must not be altered in any way. No additional printing may be set onto an article copy other than the copyright credit line required above. Any alterations must be approved in advance and in writing by AAAS. This includes, but is not limited to, the placement of sponsorship identifiers, trademarks, logos, rubber stamping or self-adhesive stickers onto the article copies.

Additionally, article copies must be a freestanding part of any information package (i.e. media kit) into which they are inserted. They may not be physically attached to anything, such as an advertising insert, or have anything attached to them, such as a sample product. Article copies must be easily removable from any kits or informational packages in which

they are used. The only exception is that article copies may be inserted into three-ring binders.

FOR CORPORATE INTERNAL USE:
The AAAS material covered by this permission may not be altered in any way. No additional printing may be set onto an article copy other than the required credit line. Any alterations must be approved in advance and in writing by AAAS. This includes, but is not limited to the placement of sponsorship identifiers, trademarks, logos, rubber stamping or self-adhesive stickers onto article copies.

If you are making article copies, copies are restricted to the number indicated in your request and must be distributed only to internal employees for internal use.

If you are using AAAS Material in Presentation Slides, the required credit line must be visible on the slide where the AAAS material will be reprinted

If you are using AAAS Material on a CD, DVD, Flash Drive, or the World Wide Web, you must include the following notice in any electronic versions, either adjacent to the reprinted AAAS material or in the terms and conditions for use of your electronic products: "Readers may view, browse, and/or download material for temporary copying purposes only, provided these uses are for noncommercial personal purposes. Except as provided by law, this material may not be further reproduced, distributed, transmitted, modified, adapted, performed, displayed, published, or sold in whole or in part, without prior written permission from the publisher." Access to any such CD, DVD, Flash Drive or Web page must be restricted to your organization's employees only.

FOR CME COURSE and SCIENTIFIC SOCIETY MEETINGS:
Permission is restricted to the particular Course, Seminar, Conference, or Meeting indicated in your request. If this license covers a text excerpt or a Full Text Article, access to the reprinted AAAS material must be restricted to attendees of your event only (if you have been granted electronic rights for use of a full text article on your website, your website must be password protected, or access restricted so that only attendees can access the content on your site).

If you are using AAAS Material on a CD, DVD, Flash Drive, or the World Wide Web, you must include the following notice in any electronic versions, either adjacent to the reprinted AAAS material or in the terms and conditions for use of your electronic products: "Readers may view, browse, and/or download material for temporary copying purposes only, provided these uses are for noncommercial personal purposes. Except as provided by law, this material may not be further reproduced, distributed, transmitted, modified, adapted, performed, displayed, published, or sold in whole or in part, without prior written permission from the publisher."

FOR POLICY REPORTS:
These rights are granted only to non-profit organizations and/or government agencies. Permission covers print and electronic versions of a report, provided the required credit line appears in both versions and provided the AAAS material reproduced as permitted herein remains in situ and is not exploited separately.

FOR CLASSROOM PHOTOCOPIES:
Permission covers distribution in print copy format only. Article copies must be freestanding and not part of a course pack. They may not be physically attached to anything or have anything attached to them.

FOR COURSEPACKS OR COURSE WEBSITES:

These rights cover use of the AAAS material in one class at one institution. Permission is valid only for a single semester after which the AAAS material must be removed from the Electronic Course website, unless new permission is obtained for an additional semester. If the material is to be distributed online, access must be restricted to students and instructors enrolled in that particular course by some means of password or access control.

FOR WEBSITES:
You must include the following notice in any electronic versions, either adjacent to the reprinted AAAS material or in the terms and conditions for use of your electronic products: "Readers may view, browse, and/or download material for temporary copying purposes only, provided these uses are for noncommercial personal purposes. Except as provided by law, this material may not be further reproduced, distributed, transmitted, modified, adapted, performed, displayed, published, or sold in whole or in part, without prior written permission from the publisher."

Permissions for the use of Full Text articles on third party websites are granted on a case by case basis and only in cases where access to the AAAS Material is restricted by some means of password or access control. Alternately, an E-Print may be purchased through our reprints department (brocheleau@rockwaterinc.com).

REGARDING FULL TEXT ARTICLE USE ON THE WORLD WIDE WEB IF YOU ARE AN 'ORIGINAL AUTHOR' OF A SCIENCE PAPER

If you chose "Original Author" as the Requestor Type, you are warranting that you are one of authors listed on the License Agreement as a "Licensed content author" or that you are acting on that author's behalf to use the Licensed content in a new work that one of the authors listed on the License Agreement as a "Licensed content author" has written.

Original Authors may post the 'Accepted Version' of their full text article on their personal or on their University website and not on any other website. The 'Accepted Version' is the version of the paper accepted for publication by AAAS including changes resulting from peer review but prior to AAAS's copy editing and production (in other words not the AAAS published version).

FOR MOVIES / FILM / TELEVISION:
Permission is granted to use, record, film, photograph, and/or tape the AAAS material in connection with your program/film and in any medium your program/film may be shown or heard, including but not limited to broadcast and cable television, radio, print, world wide web, and videocassette.

The required credit line should run in the program/film's end credits.

FOR MUSEUM EXHIBITIONS:
Permission is granted to use the AAAS material as part of a single exhibition for the duration of that exhibit. Permission for use of the material in promotional materials for the exhibit must be cleared separately with AAAS (please contact us at permissions@aaas.org).

FOR TRANSLATIONS:
Translation rights apply only to the language identified in your request summary above.

The following disclaimer must appear with your translation, on the first page of the article, after the credit line: "This translation is not an official translation by AAAS staff, nor is it endorsed by AAAS as accurate. In crucial matters, please refer to the official English-language version originally published by AAAS."

FOR USE ON A COVER:
Permission is granted to use the AAAS material on the cover of a journal issue, newsletter issue, book, textbook, or annual report in print and electronic formats provided the AAAS material reproduced as permitted herein remains in situ and is not exploited separately

By using the AAAS Material identified in your request, you agree to abide by all the terms and conditions herein.

Questions about these terms can be directed to the AAAS Permissions department permissions@aaas.org.

Other Terms and Conditions:

v 2

Questions? customercare@copyright.com or +1-855-239-3415 (toll free in the US) or +1-978-646-2777.
