# Supplementary material for: Active matter in space
Source: NPJ Microgravity. 2022 Nov 24;8:54. doi: 10.1038/s41526-022-00230-7 (PMC9700843; doi:10.1038/s41526-022-00230-7)
Supplement: Supplementary file 2 — Supplementary Information [file 41526_2022_230_MOESM2_ESM.zip › Final Permissions/Figure 1/Fig1_RMP.pdf]

American Physical Society
Reuse and Permissions License

28-Apr-2022

This license agreement between the American Physical Society ("APS") and Giorgio Volpe ("You") consists of your license details and the terms and conditions provided by the American Physical Society and SciPris.

Licensed Content Information

**License Number:**	**RNP/22/APR/053046**
**License date:**	28-Apr-2022
**DOI:**	10.1103/RevModPhys.88.045006
**Title:**	Active particles in complex and crowded environments
**Author:**	Clemens Bechinger et al.
**Publication:**	Reviews of Modern Physics
**Publisher:**	American Physical Society
**Cost:**	USD $ 0.00

Request Details

Does your reuse require significant modifications: No

**Specify intended distribution locations:**	Worldwide
**Reuse Category:**	Reuse in a journal/magazine
**Requestor Type:**	Author of requested content
**Items for Reuse:**	Figures/Tables
**Number of Figure/Tables:**	1
**Figure/Tables Details:**	Fig 1, inset on the top left part
**Format for Reuse:**	Print and Electronic
**Total number of print copies:**	More Than 10000

Information about New Publication:

**Publisher:**	Springer Nature
**Publication:**	NPJ Microgravity
**Publication Date:**	Sep. 2022
**Article Title:**	Active Matter in Space
**Author(s):**	Giorgio Volpe, Clemens Bechinger, Frank Cichos, Ramin Golestanian, Hartmut Löwen, Matthias Sperl, Giovanni Volpe

License Requestor Information

**Name:**	Giorgio Volpe
**Affiliation:**	Individual
**Email Id:**	g.volpe@ucl.ac.uk
**Country:**	United Kingdom

American Physical Society
Reuse and Permissions License

TERMS AND CONDITIONS

The American Physical Society (APS) is pleased to grant the Requestor of this license a non-exclusive, non-transferable permission, limited to Print and Electronic format, provided all criteria outlined below are followed.

1. You must also obtain permission from at least one of the lead authors for each separate work, if you haven't done so already. The author's name and affiliation can be found on the first page of the published Article.
2. For electronic format permissions, Requestor agrees to provide a hyperlink from the reprinted APS material using the source material's DOI on the web page where the work appears. The hyperlink should use the standard DOI resolution URL, http://dx.doi.org/{DOI}. The hyperlink may be embedded in the copyright credit line.
3. For print format permissions, Requestor agrees to print the required copyright credit line on the first page where the material appears: "Reprinted (abstract/excerpt/figure) with permission from [(FULL REFERENCE CITATION) as follows: Author's Names, APS Journal Title, Volume Number, Page Number and Year of Publication.] Copyright (YEAR) by the American Physical Society."
4. Permission granted in this license is for a one-time use and does not include permission for any future editions, updates, databases, formats or other matters. Permission must be sought for any additional use.
5. Use of the material does not and must not imply any endorsement by APS.
6. APS does not imply, purport or intend to grant permission to reuse materials to which it does not hold copyright. It is the requestor's sole responsibility to ensure the licensed material is original to APS and does not contain the copyright of another entity, and that the copyright notice of the figure, photograph, cover or table does not indicate it was reprinted by APS with permission from another source.
7. The permission granted herein is personal to the Requestor for the use specified and is not transferable or assignable without express written permission of APS. This license may not be amended except in writing by APS.
8. You may not alter, edit or modify the material in any manner.
9. You may translate the materials only when translation rights have been granted.
10. APS is not responsible for any errors or omissions due to translation.
11. You may not use the material for promotional, sales, advertising or marketing purposes.
12. The foregoing license shall not take effect unless and until APS or its agent, Aptara, receives payment in full in accordance with Aptara Billing and Payment Terms and Conditions, which are incorporated herein by reference.
13. Should the terms of this license be violated at any time, APS or Aptara may revoke the license with no refund to you and seek relief to the fullest extent of the laws of the USA. Official written notice will be made using the contact information provided with the permission request. Failure to receive such notice will not nullify revocation of the permission.
14. APS reserves all rights not specifically granted herein.
15. This document, including the Aptara Billing and Payment Terms and Conditions, shall be the entire agreement between the parties relating to the subject matter hereof.
